# Fluorescence Polarization Assay for Infection Diagnostics: A Review

**DOI:** 10.3390/molecules29194712

**Published:** 2024-10-05

**Authors:** Sergei A. Eremin, Liliya I. Mukhametova, Vadim B. Krylov, Nikolay E. Nifantiev

**Affiliations:** 1 Chemical Department, M.V. Lomonosov Moscow State University, Leninsky Gory, 1, 119991 Moscow, Russia; liliya106@mail.ru; 2 N.D. Zelinsky Institute of Organic Chemistry, Russian Academy of Sciences, Leninsky Prospect, 47, 119991 Moscow, Russia

**Keywords:** fluorescence polarization assay, fluorescence probes, infections, brucellosis, tuberculosis, SARS-CoV-2, oligosaccharides, peptides, nucleic acids

## Abstract

Rapid and specific diagnosis is necessary for both the treatment and prevention of infectious diseases. Bacteria and viruses that enter the bloodstream can trigger a strong immune response in infected animals and humans. The fluorescence polarization assay (FPA) is a rapid and accurate method for detecting specific antibodies in the blood that are produced in response to infection. One of the first examples of FPA is the non-competitive test for detecting brucellosis in animals, which was followed by the development of other protocols for detecting various infections. Fluorescently labeled polysaccharides (in the case of brucellosis and salmonellosis) or specific peptides (in the case of tuberculosis and salmonellosis, etc.) can be used as biorecognition elements for detecting infections. The availability of new laboratory equipment and mobile devices for fluorescence polarization measurements outside the laboratory has stimulated the development of new fluorescence polarization assays (FPAs) and the emergence of commercial kits on the market for the detection of brucellosis, tuberculosis, and equine infectious anemia viruses. It has been shown that, in addition to antibodies, the FPA method can detect both viruses and nucleic acids. The development of more specific and sensitive biomarkers is essential for the diagnosis of infections and therapy monitoring. This review summarizes studies published between 2003 and 2023 that focus on the detection of infections using FPA. Furthermore, it demonstrates the potential for using new biorecognition elements (e.g., aptamers, proteins, peptides) and the combined use of FPA with new technologies, such as PCR and CRISPR/Cas12a systems, for detecting various infectious agents.

## 1. Introduction

The emergence and spread of infectious diseases caused by viruses or bacteria have a huge impact not only on human and animal health, but also on social and economic aspects. A striking example of this was the appearance of COVID-19 with severe acute respiratory syndrome 2 (SARS-CoV-2), which led to numerous deaths worldwide and had a great negative impact on the economy [1]. The spread of infections such as avian influenza, brucellosis, etc., causes catastrophic damage to animal husbandry [2]. These diseases spread quickly among human or animal carriers through the air, water, or droplets in the environment [3,4]. Live pathogens, such as viruses or bacteria, can enter the body and cause infectious diseases. When foreign antigens enter the body, the immune system responds by producing antibodies that are specific to those antigens. To minimize the damage caused by such viral or bacterial infections, it is necessary to have simple, fast, and highly sensitive methods of diagnosing infections. Conventional clinical diagnostic strategies for infectious diseases are mainly based on pathogen culture, the detection of specific antigens, antibodies, or nucleic acids of infectious pathogens [4,5,6,7,8,9]. Serological tests, including immunoassays [9,10,11], chromatographic methods [12], or agglutination tests, and complement fixation tests [13,14] are widely used to detect biomarkers. These tests, which are usually conducted in clinical laboratories, can be time-consuming and labor-intensive. They have several general drawbacks, including high costs, dependence on expensive equipment, and the inability to obtain quick results on-site. These tests typically include multistep operations that are associated with increased cumulative errors in detection. Immune methods of analysis are often used to detect infectious diseases, such as the classic ELISA and immunochromatographic analysis (ICA). These tests have their advantages and disadvantages. ICA takes a few minutes and can be performed by anyone, but it is impossible to perform when screening a large number of samples. Heterogeneous ELISA is a labor-intensive method and is carried out in several stages. However, unlike ICA, it is convenient for conducting large-scale analysis, but often gives false results. For example, the use of nonspecific antibodies may increase the risk of false-positive results and reduce the specificity of the antigen detection. Even the most precise available-on-market ELISA tests often demonstrate false-positive results. For some agglutination tests, the specificity is less than 70 percent [13]. The aforementioned diagnostic methods have certain limitations that render them insufficient for achieving prompt, precise, on-site diagnosis during a pandemic outbreak of infectious diseases. This is particularly true in regions with limited resources, where infectious diseases are more common and severe. In the field of infectious disease diagnostics, there is an unmet need for assays with a specificity higher than 95%.

The use of homogeneous analysis, in which there is no need to separate bound and free phases, allows the development of rapid assay methods. The fluorescence polarization assay (FPA) can be a good alternative for biomarker detection in clinical disease diagnosis [15,16,17]. The FPA’s polarization fluorescence analysis method has high specificity, sensitivity, fast response, low cost, and the ability to be miniaturized, automated, and portable [18]. The design of portable instruments that can measure fluorescence polarization signals has made FPA another option for detecting infections on-site. The general advantages of FPA over the traditionally used ELISA are a simple mix-and-measure procedure and the obtaining of results within only a few minutes. FPA has been proven useful for detecting antibodies against bacterial contamination, particularly in the detection of antibodies to gram-negative bacteria [15,19]. Due to the structural features of the cell surface and the presence of antigenic lipopolysaccharides for each type of bacteria, it was possible to isolate specific oligo- or polysaccharides and obtain their fluorescently labeled derivatives. Using them as a recognition element, FPA has been successfully used for the diagnosis of bovine brucellosis and tuberculosis, demonstrating a sensitivity of 95.5% and a specificity of 99.0% for brucellosis, and values of 92.9 and 98.3% for tuberculosis, respectively [20,21]. Attempts were made to develop an FP assay for the detection of *Salmonella* bacteria, but it was not possible to achieve a high level of specificity in the assay when analyzing different strains [22]. The developed analysis for detecting the infection caused by the equine infectious anemia virus turned out to be successful; this FPA showed 100% specificity and 89.4% sensitivity [23]. A comparison of FPA and ELISA methods has shown that both methods have similar sensitivity and specificity [11,24]. However, FPA has a shorter analysis time and is easier to perform. The undoubted advantage of FPA over ELISA is the cost of the analysis, since this format requires only one reagent—a fluorescently labeled antigen.

When using FPA to detect infectious markers in biological fluid, such as serum and milk, several challenges can arise. These include a sharp change in the intensity and polarization of fluorescence, as well as the nonspecific interaction between the fluorescently labeled conjugates and medium proteins. In addition, the FP signal depends on temperature. However, matrix effect influence can be minimized by selecting buffer components. Previously, a significant drawback of the method was also the inaccessibility of equipment for measuring the FP signal. However, currently, the market offers a large number of laboratory microplate fluorimeters capable of conducting from 96 to 1536 simultaneous measurements using portable devices.

The spread of emerging infectious diseases requires rapid and specific assays that can be used at the point of care (POC) outside the laboratory. Great attention has been paid to the development of rapid assays for the diagnosis of infections after the spread of COVID-19. A promising method for rapid detection of infections at the POC is the fluorescence polarization assay, which requires only one reagent (fluorescently labeled recognition antigen), a portable device capable of measuring the FP signal, and a few minutes. The last review on the detection of infectious diseases by FPAs was published in 2003 by Jolly M.E. and Nasir M.S. [15]; more than 20 years have passed since then. However, certain advances have been made in improving the diagnosis of diseases classical for FPA detection (brucellosis, tuberculosis, etc.), and the range of detectable infections has expanded (COVID-19, avian influenza virus, etc.). This review will present the latest advances in FP assays for the rapid detection of infectious diseases in humans, animals, and even plants. Various biorecognition elements used in FPAs for the detection of infectious diseases will be considered, and it will be shown how the application of new technologies (PCR and CRISP Cas) in the FP analysis technique can contribute to the improvement of the analysis. In addition, the emergence and implementation of new materials (carbon nanomaterials, quantum dots, aptamers, etc.) in FPA diagnostics of infectious diseases can contribute to the development of highly sensitive modern methods for detecting infections, which will also be described in this review.

## 2. Principles of Fluorescence Polarization Assay

**Figure 1:**
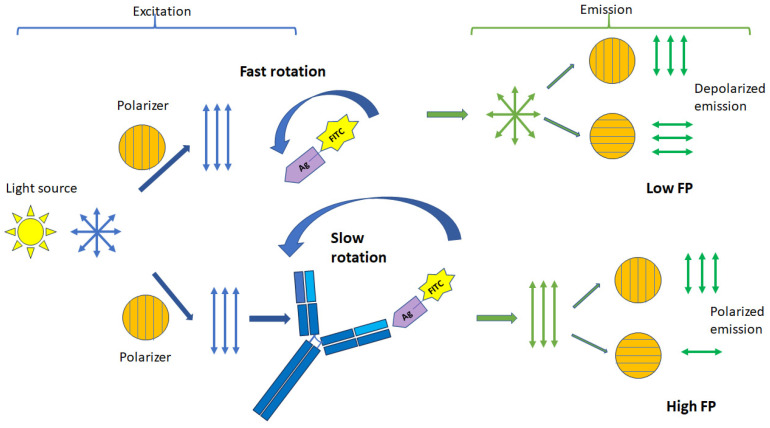
Principle of FP signal change.

Fluorescence polarization assay (FPA) may be a promising method for detecting infections in body fluid samples. The main advantages of FPA that make it useful for biomedical applications are that it is a homogeneous method that does not require complex operations such as the separation of bound and free forms of proteins, time-consuming sample preparation, and labor-intensive procedures that usually accompany clinical and biomedical analysis [18]. The principle of the FP method is described in sufficient detail in recent reviews [18,19]. The principle of measuring the FP signal is as follows: a fluorescently labeled molecule is irradiated with vertically polarized light produced by a light source with a given wavelength, then the emitted fluorescence of this molecule passes through polarization filters and is measured in the vertical (*I*_∥_*)* and horizontal (*I*_⊥_) planes [18,25] (Figure 1). These measurements are used to quantify the polarization signal (*P*). However, it is more convenient to use the millipolarization value (*mP*), where *mP* = *P* × 1000. Thus, *mP* is the difference between parallel (*I*_∥_) and perpendicular (*I*_⊥_) fluorescence intensities, normalized to the total intensity of the emitting light (Equation (1)):

(1) $$mP=\frac{I_{∥}-I_{⊥}}{I_{∥}+I_{⊥}}\times 1000$$

The value of fluorescence polarization (*mP*) depends on the hydrodynamic radius of the fluorescent compound, temperature, and viscosity [25]:

(2) $$\frac{1}{P}-\frac{1}{3}=\left(\frac{1}{P_{0}}-\frac{1}{3}\right)\left(1+\frac{RT}{\eta V}\tau \right)$$

where *P*—the observed polarization, *R*—the universal gas constant, *T*—temperature, *η*—the solution viscosity, *P*_0_—the internal polarization, *V*—the molar volume, and *τ*—the lifetime of the fluorescence excited state. The Equation (2) shows that the fluorescence polarization depends on the molecular volume (*V*) of the fluorescent molecule at fixed temperature and viscosity. Since the volume of a fluorescently labeled molecule (tracer) is proportional to the degree of FP, this can be used to detect biological processes that occur with a change in the molecular mass of a substance: binding or enzymatic cleavage of a substrate. Thus, FP can be used to measure changes in the rotational diffusion rate of a fluorescent substance when it binds to another molecule of greater molecular weight, as shown in Figure 1. In addition, these FP measurements can provide information about the size and shape of the resulting complex, which serves as the basis for quantifying the proportion of fluorophore bound to a macromolecule. FP can also be used for super-resolution optical imaging in polarization microscopy [19].

When a low-molecular-weight tracer binds to proteins or antibodies, the hydrodynamic radius increases significantly, and the *mP* value increases (Figure 1). Therefore, FPA has been found to be an excellent tool for detecting and measuring the binding of small molecules, such as fluorescently labeled antigens and hormones, to larger molecules, such as antibodies and receptors. A limitation of existing FP immunoassays is the need to use low-molecular-weight tracers. Since the change in the FP signal depends on the size ratio of the interacting molecules, it works well if the size of the labeled antigen is much smaller than the size of the interacting protein. However, the use of the non-competitive FPA format allows the development of analytical methods for the determination of large molecules. Using fluorescently labeled Fab antibody fragments, a non-competitive FPA was developed for the quantification of C-reactive protein [26]. In addition, the FPA method can also be used to detect viral particles [26,27,28] or antibodies [15]. Although these methods are useful for bioassays, their use may be limited by a high background signal or low sensitivity. Another limitation of FP arises when measuring low-affinity interactions, since in such cases, the concentration of unlabeled protein is very high, which can lead to an artificial crowding effect. The use of hydrophobic labels can lead to nonspecific interactions between molecules, and the fluorescent dye can change the binding properties of the antigen. It is important to note that the FP signal is sensitive to changes in temperature and solution viscosity. However, due to the specificity, speed, ease of analysis, the introduction of new portable instruments, and the emergence of new materials, FPA is the most promising analysis technique at the point of care (POC).

## 3. Detection of Infectious Diseases by FPA

**Figure 2:**
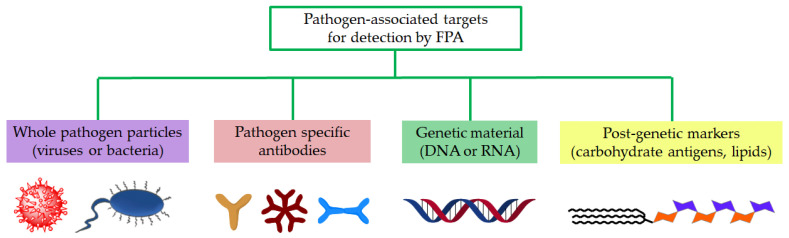
Targets used for diagnostics of infectious diseases.

Infectious diseases are defined as disorders caused by pathogenic microorganisms: bacteria, viruses, parasites, or fungi. Despite advances in the treatment of infectious diseases such as pneumonia, influenza, tuberculosis, HIV/AIDS, malaria, etc., they continue to cause millions of deaths worldwide and remain a pressing challenge for public health systems. Rapid diagnostic tests are needed to detect infectious diseases. Such tests should be simple and relatively quick to perform so that they can be used not only in the clinic but also at the point of care (POC). FPA is applicable for the selective detection of a variety of pathogen-associated markers. They include all possible biomacromolecules expressed by viruses, bacteria, fungi, and other pathogens—nucleic acids (DNA and RNA) and post-genomic markers (proteins, glycoconjugates, lipids etc.) as well as the antibodies produced against the above-listed markers (Figure 2).

**Figure 3:**
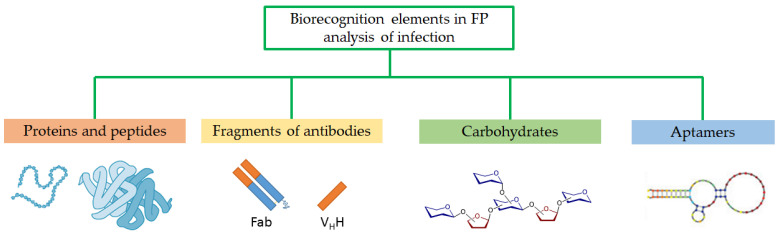
Biorecognition elements used in FPA for detection of infectious diseases.

FPA methods have been used in the clinical laboratory to diagnose infectious diseases for more than four decades [15,27,28]. The non-competitive FPA format is primarily used to detect infectious diseases, since this method is based on the detection of large molecules: antibodies, nucleic acids, and, in rare cases, the bacteria themselves. Therefore, it is preferable to use an antigen with a small molecular weight (no more than 20–30 kDa), for example, oligosaccharides, peptides, small proteins, or aptamers, as a recognition antigen (Figure 3). Fluorescein is mainly used as a fluorescent label, but currently, there are a large number of different dyes on the market with different excitation and emission wavelengths, which allows one to select a dye for any task. The increase in FP signal of the fluorescently labeled antigens after specific binding to antibodies or nucleic acids can be used to develop a sensitive assay. Based on the advantages of the method outlined above, FPA has all the potential for successful application in infection detection.

## 4. Detection of Antibodies to Viruses and Bacteria by FPA

The diagnosis of infectious diseases is often based on the determination of specific antibodies. Bacteria entering an animal or human body cause an immune response and the formation of specific antibodies. It is known that gram-negative and gram-positive bacteria have different cell membrane structures. The cell wall of bacteria is made up of several glycan structures that cover the cells. Based on the composition of their cell walls, most bacteria fall into one of three categories: mycobacteria, gram-positive bacteria, or gram-negative bacteria [29]. Gram-negative bacteria are characterized by an outer membrane that is associated with capsular polysaccharide (CPS) and lipopolysaccharide (LPS), an intermediate periplasmic space, and peptidoglycan [30]. Both gram-positive and gram-negative bacteria have capsular polysaccharides and a thick coating of peptidoglycan on their cell surfaces. On the other hand, teichoic acids rather than LPS are produced on the surface of gram-positive bacteria, which have a single cell membrane [31]. Mycobacterium tuberculosis, a common human pathogen, is one of the bacteria with distinct cell walls that allow them to be classified as their own category [31,32]. LPS, or endotoxin, can cause endotoxic shock, tissue injury, sepsis, multiple organ failure, and death [33]. In addition, it is extremely immunogenic and induces the formation of specific antibodies that are used to detect infectious diseases [15]. Fluorescence polarization assay (FPA) has the potential to replace more expensive serologic tests. The assay has been validated for brucellosis in many animal species, and in various serum, whole blood, or milk samples, making FPA more versatile than other tests. As shown in Table 1, FPA has demonstrated high sensitivity and specificity in diagnosing infectious diseases and can be used to diagnose other infections.

### 4.1. Antibodies to Brucellosis

A classic example of the detection of bacterial infections in veterinary care by FPA is the detection of brucellosis in cattle [15]. The most significant bacteria from a public health standpoint are *Brucella melitensis* and *Brucella abortus*, which typically infect livestock and cattle, respectively. Early diagnosis is the basis for the prevention, treatment, and control of brucellosis. Although isolation and cultivation of bacteria are currently the gold standard for detection of brucellosis [34], serological diagnosis of brucellosis, which is simpler and faster, is widely used to diagnose the disease [41]. The Rose Bengal Test (RBT) and the Serum Agglutination Test (SAT) serve as confirming examinations for the diagnosis of brucellosis [14,42,43,44]. These confirmatory tests do have somewhat involved and time-consuming procedures, and because false-negative results can occasionally occur, subjective factors can easily influence how the results are interpreted. More trustworthy tests for the diagnosis of brucellosis are obviously needed. Based on antigen–antibody interactions, the fluorescence polarization assay (FPA) is a reasonably quick and accurate way to detect antibodies or antigens [15,45]. LPS can be easily purified from gram-negative bacteria and hydrolyzed, and its OPS fragment can be used as a biorecognition element to detect specific antibodies. FP assays using fluorescein-labeled OPS derived from *Brucella abortus* are known to be used to detect antibodies to *Brucella* in animals [15] and humans [16,45,46] (Table 2). FPA has been approved as a laboratory test for animal brucellosis and satisfies the requirements set forth by the World Organization for Animal Health. FPA has the benefit of having a 5 min reaction time and being usable for both large-scale field screening and individual diagnosis [30].

Some studies have reported that FPA is widely used to detect antibodies to *Brucella* spp. in serum and the whole blood of cattle [34], sheep [17,36], pigs [37], cervids [38], camels [47], and other animals. This method is also used to detect antibodies in milk [35] (Table 2). There are also several reports of the detection of brucellosis in humans using FPA [15,48]. Usually, the cut-off value for FPA is calculated as 5 standard deviations above the negative control and is typically 10 *mP* delta (a sample whose signal is 10 *mP* above the negative control is considered weakly positive). Typically, such an FP signal is detected in animals that have already had brucellosis or in vaccinated animals, and an FP signal that exceeds the negative control by 20–40 *mP* is detected in animals in the acute phase of the disease [15].

Ellie (Germantown, WI, USA) produces a kit for determining brucellosis in cattle and humans using the FPA method [49], which detects antibodies against *Brucella* in serum. As the biorecognition element, this test also uses O-chain polysaccharide antigen (OPS) extracted from *B. abortus* and *B. melitensis*, conjugated to fluorescein. The presence of antibodies specific to the OPS antigen indicates a current or recent brucellosis infection. Compared to other *Brucella* methods, FPA measures the antibody response against the OPS portion of lipopolysaccharide (LPS), while RBT, ELISA, and most cELISA kits detect antibody responses to all constituent parts of LPS, including lipid A and the core oligosaccharide. It is important to note that the FPA test is a semi-quantitative system for measuring *Brucella* activity in animals, allowing for the distinguishing of animals infected with field strains or vaccines from animals without infection [50].

A systematic review and meta-analysis were conducted to evaluate the accuracy of serological tests used in the diagnosis of bovine brucellosis, and estimated the diagnostic sensitivity (DSe) and diagnostic specificity (DSp) [51]. The databases CABI, Cochrane Library, PubMed/MEDLINE, SciELO, Scopus, and Web of Science were used for data selection, and 178 analyses and 11 different serological tests were considered. The best DSe and DSp were demonstrated by the iELISA (indirect enzyme immunoassay with bacterial suspension as antigen— 97.7 and 99.97, respectively) and FPA tests (97.7 and 99.97, respectively), and FPA also showed the best overall accuracy (99.71). Thus, the advantages of FPA for detection of brucellosis both inside and outside the laboratory compared to other serological tests were demonstrated [51].

### 4.2. Antibodies to Salmonella

Another infectious disease that requires intense control efforts and continues to be a persistent and costly public health problem on several continents is caused by the bacteria *Salmonella enteritidis,* which is transmitted to humans through eggs [6,52]. As with the confirmation of brucellosis, birds suspected of being infected with *S. enteritidis* are tested by culturing environmental samples [6] to detect the presence of the bacterium. This is followed by confirmation using other methods, such as agglutination and ELISA. The development of immunoassays based on lipopolysaccharide antigens makes it possible to effectively detect infected birds (Table 2). The successful application of the FPA method for the detection of antibodies to *Brucella* was extended to the diagnosis of infections caused by other gram-negative bacteria with OPS. FPA was developed to determine antibodies to some types of salmonella in chicken serum and egg yolks [15,21,53]. Because eggs can be collected with minimal difficulties without causing stress to laying hens or compromising the biological safety of the birds, they are attractive samples for antibody testing. *Salmonella* polysaccharide antigens vary among different serotypes and serogroups, making it difficult to interpret the results of serological tests. However, a high degree of specificity for differentiating *S. enteritidis* and *S. typhimurium* infections could not be demonstrated. Fluorescently labeled OPS from different bacterial strains did not show any clear or consistent pattern of sensitivity and specificity. Since *Salmonella* antibody tests are currently of great interest in identifying potentially infected birds for large-scale egg production, specificity is less important than sensitivity. However, the lack of specificity of serological screening tests can result in wasted resources when subsequent egg culture is performed in flocks infected with *Salmonella* serotypes that are antigenically cross-reactive but of unknown public health significance. Similarly, *S. typhimurium* infection could be reliably detected by fluorescence polarization with an *S. typhimurium* tracer, but samples from hens infected with *S. enteritidis* also tended to cross-react with the tracer. Although issues with specificity may restrict the utility of antibody test results, fluorescence polarization seems to provide a quick and easy substitute for traditional serological techniques [21].

### 4.3. Antibodies to Tuberculosis

Tuberculosis is another important disease in veterinary medicine. Mycobacterium bovis, a member of the Mycobacterium tuberculosis complex, is the causative agent of bovine tuberculosis (bTB). The prevalence of this illness is high in low- and middle-income nations. The comparative intradermal (CID) test, also known as the skin test, which utilizes protein derivatives (PPD), is the gold standard for bTB diagnosis. The CID and other cell-mediated immune tests (e.g., interferon-gamma (IFN-γ) and lymphocyte proliferation tests) have many limitations in diagnosing bTB. The limitations of these methods are the low sensitivity and specificity in measuring delayed-type hypersensitivity in response to antigens.

The structure of *M. bovis* bacteria is very different from that of gram-negative and gram-positive bacteria [31,32]. To develop an FPA for detecting tuberculosis, a different strategy was chosen. Proteins and peptides were used as biorecognition elements in FPA. Currently, the most well studied mycobacterial antigens are MPB70 and MPB83 [54,55]. They are highly homologous proteins within *M. tuberculosis* complex members. They are major antigens highly expressed by *M. bovis* and considerably less abundantly expressed by *M. tuberculosis* [32].

The protein MPB70 is secreted by *M. bovis,* with subsequent degradation to a peptide consisting of 30 amino acids [54] (Table 2). A fluorescently labeled MBP70 protein conjugate was used to develop FPA for tuberculosis, and initially, when testing a large number of serum samples from sick and healthy animals, 92.9% sensitivity and 98.3% specificity were shown; however, when comparing animal samples with different stages of the disease, the sensitivity of the method dropped to 50%, less than that of the ELISA test [15,21,39,54]. It turned out to be possible to solve this problem by using not the native protein, but its fragments, as recognition elements. The 96 peptides of 15 amino acids were synthesized, and in the last stage of synthesis, fluorescently labeled derivatives were prepared, purified by HPLC, and tested for binding to tuberculosis-positive sera. As a result, a peptide was selected, and a sensitive method for detecting tuberculosis in animals was developed. Currently, based on this peptide, the TB FPA kit [56] is an examination of quality that assesses the presence of antibodies against *M. bovis* in bovine serum using a fluorescence polarization assay. The test may be run in the field or in the lab using portable fluorescence polarization equipment. The purpose of TB antibody tests is to identify anergic animals that are nonresponsive to skin probes or gamma-interferon release assays.

The ESAT-6 protein, which has a mass of 6 kDa and is generated by *M. tuberculosis*, was chosen as another antigen [40]. Fluorescein-labeled ESAT-6 protein is used as a biorecognition element in the FPA test, which assesses the humoral immune response. The binding of the fluorescent-labeled conjugate of ESAT-6 to antibodies against bacteria leads to an increase in fluorescence polarization. CID skin reaction and IFN-γ screening were used as markers of tuberculosis in cattle. The IFN-γ test is highly specific, but it is very expensive, which leads to certain limitations. A cut-off value of greater than >127 *mP* offered the best discrimination between positive reactors and negative bTB animals, based on the *mP* values from five culture-positive serum samples. When comparing the FPA results using ESAT-6 as the recognition element with the CID results, the FPA showed a sensitivity of 92.9% and a specificity of 64.6%, while the IFN-γ results showed a sensitivity of 95.7% and a specificity of 49%. When it comes to humoral immune response in animals, FPA with FITC-labeled ESAT-6 as a tracer has superior sensitivity (95.7%) and specificity (49.1%) to that of the IFN-γ test. Thus, FPA has the ability to detect antibodies in cattle infected with *M. bovis.* The use of ESAT-6 in FPA helps to identify infected animals that are not detected by CID and IFN-γ tests, especially in herds with a low infection rate. Another piece of evidence confirming the high specificity and sensitivity of the protein is that, despite the presence of M. bovis infection in some herd animals, no skin reactions were observed. However, a negative test result for CID and IFN-γ does not mean that the animal is not infected; it simply means that it does not shed [40].

### 4.4. Antibodies to Equine Infectious Anemia Virus (EIAV)

Equine infectious anemia (EIA) is a potentially fatal blood-borne viral disease [57]. Despite the fact that most equids that are infected today do not show external signs of the disease, testing is recommended when animals are on the move or in large gatherings. This illness has no known cure or vaccine, and it can be challenging to differentiate EIA from other fever-producing illnesses like equine encephalitis, anthrax, and influenza. People are also affected by the disease; these individuals frequently develop a chronic form of the illness, characterized by frequent fever episodes and other clinical symptoms like swelling, weight loss, and severe anemia. While the virus is always present in the tissues of horses suffering from the chronic form of the disease and makes them contagious to other horses, horses suffering from the clinical form of the disease have significantly higher blood virus concentrations.

Equine infectious anemia virus (EIAV) infections have been controlled in horses by identifying and removing seropositive animals in centralized laboratories using a standardized agar gel immunodiffusion (AGID) test [58]. Immunoassays developed for detection of EIA are known [59,60]. The current lack of rapid diagnostic methods that are easily applied in the field is impeding the screening of horses that test positive for EIAV. Based on FPA, a quick solution test for EIAV antibodies has been created [23,41] (Table 2). For use as a probe in FPA and as a peptide antigen, a peptide (corresponding to the immunodominant region of the EIAV transmembrane protein) derived from the antigenic regions of the EIAV core and envelope proteins was chosen, and its fluorescent derivative was obtained. The interaction of this probe was examined in an enhanced FP assay employing AGID-positive and AGID-negative equine serum. These investigations’ findings demonstrated that the FP assay’s reactivity has 100% specificity, 89.4% sensitivity, and the ability to readily identify antibodies generated during the initial phases of infection. Ellie’s EIA FPA is a qualitative test that looks for antibodies against the equine infectious anemia virus in horse serum samples [61]. The presence of antibodies indicates EIAV infection. A peptide conjugate is used as a tracer in the diagnostic test. Antibodies against the EIA virus can be found using the straightforward, fast, and precise EIA FPA assay. This fluorescence polarization assay, which is based on synthetic peptides, finds antibodies that are specific to the viral gp45 protein [61]. It is advised to use this non-subjective test as a field assay or as a substitute for AGID assays.

### 4.5. Antibodies to Avian Influenza a Virus (IAV)

Animal diseases known as transboundary animal diseases (TADs) are extremely contagious and are spreading quickly around the world [62,63]. Pathogen eradication has become a challenge as a result of TADs spreading across the nation, causing severe economic losses to the livestock industry. Rapid testing techniques are required to prevent the spread of infected animals with TAD and economic damage [62,63].

Highly pathogenic avian influenza A virus (IAV), known as bird flu, is proliferating globally as a result of the growing poultry trade between countries and the migration of wild waterfowl across borders. Based on the antigenicity of the surface glycoproteins neuraminidase (N1–N9) and hemagglutinin (HA; H1–H16), IAVs are divided into several subtypes. It is commonly known that certain virus subtypes, like H5N1, can infect humans who live with or work closely with birds, as well as cause serious illness and high mortality rates in the poultry industry. As a result, it is critical to monitor the avian IAV subtypes H5 and H7. The creation of a quick testing technique to identify the virus and antiviral antibodies in infected animals is required to stop the spread of these virus subtypes. FPA is a convenient tool to discover the anti-H5 IAV avian antibody in serum. Dual testing of antibodies to all subtypes and H5- or H7-specific subtypes is required to strictly control highly pathogenic avian influenza. Furthermore, early detection of viral antigens of the H5 or H7 subtypes is essential in the event of avian IAV infections. A quick and easy protocol was created for the detection of IAV-related antibodies by FPA [64].

The influenza virus attaches itself to susceptible cells through the viral protein hemagglutinin (HA), which identifies glycoconjugates on the cell surface that end in α-sialosides. With fluorescence polarization immunoassay (FPIA), anti-H5 subtype IAV antibodies in serum can be quickly, easily, and selectively detected [64,65]. To be used as a labeled antigen in FPIA, a fragment of recombinant H5 subtype IAV hemagglutinin was created and labeled with fluorescein. Using a portable FP analyzer, the labeled antigen was combined with anti-IAV sera (H1–H16 subtypes), and the mixture’s FP was measured. It was found that the FP measured for anti-H5 IAV serum was significantly higher than that obtained for other H-types’ sera (anti-H1–H16, except anti-H5), and increased proportionally with the concentration of the anti-H5 antibody. It was verified that the anti-H5 subtype IAV antibody could be detected selectively. The original sample volume required was 2 μL, and the analysis time was limited to 20 min [65]. Effective on-site diagnosis and surveillance of IAV is made possible by this detection system.

### 4.6. Antibodies to SARS-CoV-2

The COVID-19 pandemic has led to increased attention on methods for detecting antibodies to viral infections [66]. The current gold standard for COVID-19 detection is a molecular test, but because these are done in central laboratories, decisions about treatment and control are delayed. In addition, there remains an ongoing need to quantify the immune response for the development of vaccines and treatments for COVID-19. Analytical techniques have been developed for the quick and quantitative detection of antibodies to SARS-CoV-2 in human serum using fluorescence polarization immunoassay (FPIA), in order to rapidly establish the diagnosis and immune status of patients. FPIA was performed using recombinant SARS-CoV-2 receptor binding domain (RBD) protein labeled with HiLyte Fluor 647 (F-RBD). The degree of polarization (P) rises when anti-RBD antibodies in human serum attach to F-RBD, preventing F-RBD from diffusing in rotation. All that was needed for the measurement process was to combine the F-RBD reagents with the serum sample, and then, after a 15 min incubation period, measure the P value using a portable fluorescence polarization analyzer. The FPIA system demonstrated high accuracy (AUC = 0.965) in identifying serum that was either COVID-19 positive or negative. The measurement took approximately 20 min to complete and 1 μL or less of serum was needed. Thus, the findings showed that the FPIA method has a high throughput, fast turnaround time, great accuracy, and ease of use for diagnosing COVID-19. Comparatively speaking, current quantitative ELISA techniques for measuring anti-COVID-19 virus antibodies with comparable AUC values take 1–2 h and necessitate 3–5 assay washing procedures [66,67,68]. Candidate threshold values calculated from the ROC curve (Δ*mP* = 0.83 or 2.17) were proposed, which provided an analysis with maximum sensitivity and specificity (Se/Sp 100/100 and 80/80%, respectively). High overall accuracy, 100% sensitivity, 98.4% specificity, and 98.8% agreement are provided by the designed ELISA. The effectively created FPIA system makes it possible to quickly and simply quantify antibodies against SARS-CoV-2. This FPIA test system will enhance knowledge of the immune response to COVID-19 and enable quick on-site identification of infected individuals [28].

**Table 2:** Summary of detection of antibodies to bacteria and viruses by FPA.

Disease	Sample	Fluorescent Recognition Element	Ref.
Brucellosis	Bovine sera	Flu-OPS	[20,34,47,49] *
	Milk	Flu-OPS	[35]
	Sheep sera	Flu-OPS	[17,36]
	Pig sera	Flu-OPS	[37]
	Cervid sera	Flu-OPS	[38]
	Camel sera	Flu-OPS	[46]
Salmonellosis	Egg yolk Chicken sera	Flu-OPS	[21] [53]
Tuberculosis	Bovine sera	Flu-MPB70	[21,39]
	Bovine sera	Flu-ESAT-6 protein	[40]
	Bovine sera	Flu-peptide	[54,56] *
Paratuberculosis	Bovine sera	Flu-protein fraction of 35 kDa from Map 3065	[24]
Equine infectious anemia virus	Horse sera	Flu-peptide	[23,41,61] *
Influenza A virus	Chicken and goat sera	Flu-peptide	[58]
	Chicken and goat sera	Recombinant H5 subtype HA Flu-proteins	[65]
SARS-CoV-2 Antibody	Human sera	SARS-CoV-2 receptor binding domain (Flu-RBD) protein	[28]

* This is the reference corresponding to the commercial FPA kits.

## 5. Detection of Virus Particles with the Use of FPA

**Figure 4:**
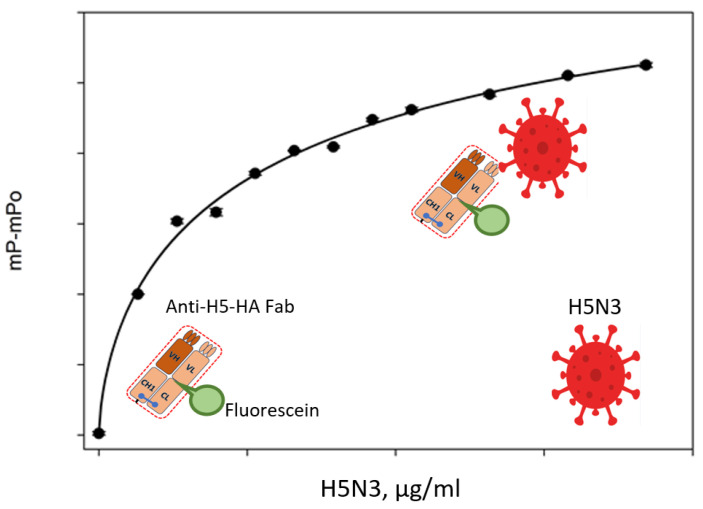
Scheme for detection of H5N3 viruses using fluorescently labeled Fab fragments of antibodies by the FPA method.

Viral infection diagnosis is getting a lot of attention these days. The ability to detect viruses with FPA was demonstrated using the example of detecting H5 avian influenza virus using a portable analyzer. A separation-free immunoassay for virus detection called the non-competitive fluorescence polarization immunoassay (NC-FPIA) was developed. The proof of concept employed the H5 subtype avian influenza virus (H5-IAV) as a model virus. The fluorescein-labeled Fab fragment, which binds to hemagglutinin H5 (HA), was used for NC-FPIA. The fluorescein-labeled Fab fragment was combined with the purified H5-IAV, which contains H5 HA, and using a portable FPIA analyzer, the degree of fluorescence polarization was determined (Figure 4). A 15 min incubation period was sufficient to detect H5-IAV. Furthermore, NC-FPIA can be performed on-site using a sample volume of no more than 20 μL due to the portable FPIA analyzer. This is the first study where an FPIA virus particle has been found. One can use this NC-FPIA to quickly diagnose different viruses on the spot [27].

The attachment of influenza virus to susceptible cells is mediated by the viral protein hemagglutinin (HA), which is located on the virus surface and recognizes cell surface glycoconjugates terminated with α-sialosides. A new class of fluorescent glycocluster nanoparticles (quantum dots (QDs)) with sialylated N-glycan chains (A2-PC-QDs) that bind to HA was designed and obtained for the development of anti-influenza drugs. FPA allowed competitive analysis of A2-PC-QDs binding to HA in the presence of potential low-molecular-weight inhibitors. It was shown that A2-PC-QDs efficiently bind to HA, and therefore, FPA based on these QDs is a useful tool for high-throughput screening and for acceleration in the development of new and more effective blockers of influenza virus attachment, and that FP method can be used to detect viral particles [69].

Due to easily programmable molecular recognition and predictable local geometry, DNA has been recognized for more than 30 years as a useful building block for both nanotechnology and signal amplifiers [70]. Structural DNA is an ideal FP amplifier for fluorescence probe binding and release-based FP modulation because of its high molecular weight, well-organized geometric structure, and low absorption in the long wavelength range [70]. DNA is a highly promising tool for the development of FP-based biosensors which combines DNA structural nanotechnology with isothermal nucleic acid amplification to regulate the FP signal change [71,72]. This made it possible to develop FP-sensitive circular isothermal strand displacement amplification (FP-CSDA) for Salmonella detection on multivalent DNA monolayers attached to a hairpin aptamer probe (MHAP-DNA monolayers) [52]. The MHAP-DNA monolayers in this system were built using a dsDNA tile-directed self-assembly mechanism. The signaling unit is a FAM-labeled reporting probe (RP-FAM) that has a built-in low FP signal. When target Salmonella is present, MRP-FAM is trapped in the super DNA monolayers by means of target-triggered CSDA, which then removes the tethered hairpin-structured aptamer probes (HAPs) that are in charge of binding RP-FAM. Because the target Salmonella can be recycled and structural DNA materials have the ability to strongly restrict the free rotation of the FAM fluorophore without having an effect on fluorescence quenching, the FAM fluorophore’s FP signal can be amplified significantly. The findings of the experiment show that the FP assay has high specificity and a low limit of detection (LOD) of 7.2 × 100 CFU/mL for Salmonella. Thus, the possibility of using DNA nanoarchitecture as a basis for modulating CSDA-based FP assays for bacterial detection is demonstrated.

## 6. Detection of Nucleic Acid with the Use of FPA

The FPA method has been successfully used for the rapid detection of nucleic acids, providing an efficient and cost-effective way to detect infectious diseases in humans and plants [9]. The increase in microbial resistance demands the development of new, simple, and rapid platforms for the detection of infections. In this context, the FPA can be regarded as the method of choice for many cases. Particularly, the natural adaptive immune system of bacteria, known as clustered regularly interspaced short palindromic repeats (CRISPR) and CRISPR-associated protein (Cas), has been developed as a tool to perform genomic edits in any genome of interest, including that of microbes and humans. As a result, numerous studies have been conducted to apply this technology to detect infectious diseases and improve diagnostics, and to monitor the development of antibiotic resistance and the efficiency of the treatment of persistent infections [73,74].

Currently, the CRISPR/Cas12a system is a powerful tool known for its high specificity in DNA analysis; in combination with fluorescence polarization, it can significantly reduce the detection limit (detect only a few copies of DNA in the sample) and detect infection at the very beginning, when antibodies have not yet formed, or when this is completely impossible, for example, when detecting bacterial infection of plants. A portable assay system was described that allows for quick on-site COVID-19 diagnosis. Known as CODA (CRISPR Optical Detection of Anisotropy), the technique eliminated unnecessary manual steps by combining isothermal nucleic acid amplification, CRISPR/Cas12a activation, and signal generation in a single assay. Significantly, the ratiometric measurement of fluorescent anisotropy served as the basis for signal detection, enabling CODA to attain a high signal-to-noise ratio. A small, stand-alone CODA device with an embedded heater, optoelectronics, and a microcontroller for data processing for point-of-care applications was developed. After loading the sample, the developed system finished detecting SARS-CoV-2 RNA in 20 min, with a detection limit of 3 copies/μL. The rapid CODA test correctly identified COVID-19 status when applied to clinical samples (10 confirmed COVID-19 patients; 10 controls), in line with gold standard clinical diagnostics [75]. For COVID-19 diagnostics, CRISPR systems have shown to have promising potential. However, two technical issues prevent these assays from being used in a POC setting: (1) most CRISPR tests involve sequential NA amplification and detection, necessitating the preparation and addition of CRISPR reagents into samples; and (2) signal readout, which is usually done with a dipstick-type lateral flow device, adds additional manual steps and yields subjective, qualitative results.

With the emergence of miniature PCR devices on the market [76,77], nucleic acid testing is a promising tool for the sensitive, specific diagnosis of infections. Most often, a fluorescent signal is determined, which measures the accumulation of a PCR product either in real time or at the end of a given amplification reaction. The analytical signal is usually obtained using intercalating dyes (for example, SYBR green I), which begin to fluoresce upon binding to double-stranded DNA [76]. The method is quite simple and economical, and only one reagent is used to generate the signal. However, it may be susceptible to false-positive results due to the formation of a primer dimer. The newly developed PCR scheme makes it possible to identify pathogens quickly and efficiently. The method uses three main components: a detector probe, a universal DNA reporter conjugated with a single fluorophore, and FP detection. The developed approach has a number of advantages over conventional PCR protocols: (1) the analysis is simple and fast; (2) the separation of target recognition (detecting probe) and signal generation (universal reporter) reduces the cost of analysis while maintaining high specificity; and (3) the detection of FP is resistant to environmental factors (e.g., pH, photobleaching, temperature, opacity) and can be performed in a simple way in a homogeneous analysis format. As an illustration of this concept, FP analysis was used to detect *Salmonella* infection. Since multiple copies of bacterial mRNA would increase the likelihood of detection, bacterial mRNA was selected as the main target. Prior to amplifying the target mRNA using an asymmetric reverse transcription (RT-) PCR, total RNA was extracted from the specimen. The all-in-one master mix with detection and universal reporter probes was then added. Reusing the DNA polymerase for the extension reaction on the reporter probes allowed it to maintain its activity during the RT-PCR. As a result, all of the steps could be completed in one tube without the need for washing. After an incubation phase, the degree of fluorescence polarization (FP) was assessed. Due to using 30-mer sequences with maximum specificity to the target Salmonella DNA, a detection sensitivity of ~1 colony-forming unit (CFU) in blood samples was achieved, and the different strains were subtyped based on their genetic differences [9].

Currently, different methods for diagnosis of human viruses are applied. For diagnosis of human immunodeficiency virus (HIV), antibody tests, antigen/antibody tests, and nucleic acid tests (NATs) are used. The hepatitis B virus (HBV) is determined using three main markers: hepatitis B surface antigen (HBsAg), antibody to hepatitis B surface antigen (anti-HBs), and antibody to hepatitis B core antigen (anti-HBc). Detection of antibodies is possible only a month after infection, which is not suitable for early diagnosis. The viral nucleic acids are the earliest indicators after virus invasion. An FP platform has been developed for screening HIV and HBV. HIV-DNA detection based on T7exonuclease-assisted target recycling amplification with graphene oxide (GO) was developed [78]. This method achieved a detection limit of 38.6 pM. The use of dendrite-modified gold nanoparticles as an amplifier of the FP signal contributed to the achievement of a detection limit for HIV-DNA detection of 73 pM [79]. 

Hepatitis B virus (HBV) has been divided into eight genotypes (A to H) based on sequence divergence in the genome. Individuals infected with different genotypes of HBV have different responses to drug therapy and different risks of recurrence and side effects. A method for identifying HBV genotypes A to D using an FP assay involving asymmetric PCR and hybridization is presented [80]. Thus, the platform developed on the basis of FP is capable of not only determining HBV-DNA but also determining the genotype, which is important for prescribing treatment and predicting the severity of the disease.

**Figure 5:**
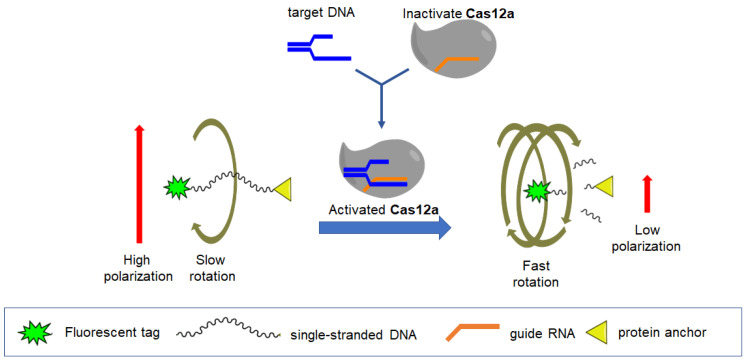
A schematic representation of the FA assay based on CRISPR/Cas12 using a single-stranded DNA probe with a protein anchor [82].

For the identification of infecting plant pathogens, it is more convenient to use DNA detection. CRISPR/Cas12a technology has attracted increasing attention as a tool for specific DNA recognition. Cas12a endonucleases in a complex with guide RNA (gRNA) are able to initiate two sequential processes: (1) recognition of the nucleotide sequence of the target dsDNA and complementary gRNA, and (2) activation of the trans-nuclease ability of Cas12a (trans-cleavage) against any single-stranded DNA (ssDNA) at least five nucleotides in length [81]. This system has a number of advantages because it is capable of detecting the DNA of pathogens, which is important at the very beginning of infection or when detecting antibodies is impossible. An identifying method of *Erwinia amylovora* DNA is presented [82]. *Erwinia amylovora* is the causative agent of bacterial blight of fruit crops, a disease that affects most plant species of the subfamily Maloideae of the rose family Rosaceae (Spiraeoideae). A simple and versatile approach has been proposed to convert conventional Cas12a-cleavable DNA probes into improved tools for measuring fluorescence anisotropy (FA). Linear and hairpin ssDNA probes, each with fluorescein at one end and a spinning tool (anchor) at the other, were studied. Conditions were selected, and the optimal set (probe structure, anchor, divalent ion concentration) was determined for the most sensitive analysis and specific recognition of double-stranded DNA (dsDNA) fragments of the bacterial phytopathogen *Erwinia amylovora* using Cas12a (a schematic diagram of the analysis is presented in Figure 5). The detection limit of the target dsDNA was 0.8 pM, which is eight times more sensitive compared to the conventional fluorescence-based method. The enhanced kit provided detection of single *E. amylovora* cells per reaction in a CRISPR/Cas12a-based recombinase polymerase amplification assay. This approach is universal and easy to implement, and the combination of fluorescence anisotropies with Cas12a can provide increased sensitivity and reliability of the signal and can be applied to various DNA and RNA analytes. Thus, new technologies such as PCR and CRISPR/Cas in combination with fluorescence polarization can significantly increase the sensitivity of the FPA method for detecting nucleic acids of viruses and bacteria, and eliminate nonspecific binding and background signals.

## 7. Conclusions and Future Trends for the Use of FPA for Infection Detection

The spread of infectious diseases caused by viral, bacterial, fungal, and other pathogens is alarming worldwide. For the rapid detection of diseases and their localization, fast, sensitive, and portable analytical methods are required that can be carried out on the spot. Classical methods for detecting infections are relatively expensive, time-consuming, and require specialized laboratories. The fluorescence polarization assay has been successfully used for many years to develop competitive FPA formats for the determination of small molecule analytes (herbicides, antibiotics, mycotoxins, etc.).

Currently, a number of commercial tests already exist on the market and are used for the detection of dangerous diseases in humans and animals, including brucellosis, tuberculosis, and equine infectious anemia virus, and the list will only expand. For detecting infectious markers, use of the FPA method requires solving some problems: (1) when analyzing biological fluids with a complex composition (milk or blood serum), nonspecific interaction of matrix proteins with fluorescently labeled recognition elements can lead to a noticeable increase in polarization and fluorescence intensity, which contributes to the occurrence of false-positive results; (2) difficulties can arise when testing vaccinated and infected animals; and (3) cross-reaction with other pathogens is possible due to the similarities in their structures, which reduces the specificity of the test. However, modern technologies provide a large number of options for solving these problems. The use of fluorescently labeled Fab fragments of antibodies, recombinant nanobodies, synthetic oligosaccharides, aptamers, small proteins, or peptides in non-competitive analysis formats opened the way to expand the scope of application of FPA for the determination of big molecular analytes: enzymes, proteins, antibodies, viruses, bacteria, cells, and others. These capabilities are successfully used in the development of new, fast, sensitive, and specific methods for the detection of infectious diseases. The use of new technologies in the CRISPR/Cas12a system, together with fluorescence polarization, makes it possible to determine the specific DNA of bacteria or viruses in samples. In contrast to antibody testing, DNA detection allows for the detection of bacterial or viral infection at an early stage, when antibodies have not formed yet, as well as for the examination of samples in which antibody detection is not possible (water, food products, plant extracts, etc.). Another promising development of FPA for the rapid detection of pathogenic bacteria becomes possible when using aptamers as recognition elements. Aptamers are short sequences of single-stranded DNA or RNA that can selectively bind to target molecules. They are stable, easily subject to chemical modification, have a small size, are non-immunogenic, and can be obtained for almost any target. However, the process of obtaining aptamers for identifying bacteria may encounter several difficulties, which may be associated with the size and complex surface structure of bacteria that complicate the selection process [83]. A simple and rapid fluorescence polarization (FP) platform was established for the detection of *Weissella viridescens* (a meat spoilage bacterium), in which a FAM-tagged complementary sequence (FAM cDNA) was used to generate the FP signal, and streptavidin was used to amplify the FP signal. Under optimal conditions, *W. viridescens* concentration and FP value had a good linear relationship with a detection range of 10^2^ to 10^6^ CFU/mL [84]. Thus, the use of FPA in bacterial recognition has significant advantages over traditional methods, and the use of aptamers will only contribute to its spread.

**Figure 6:**
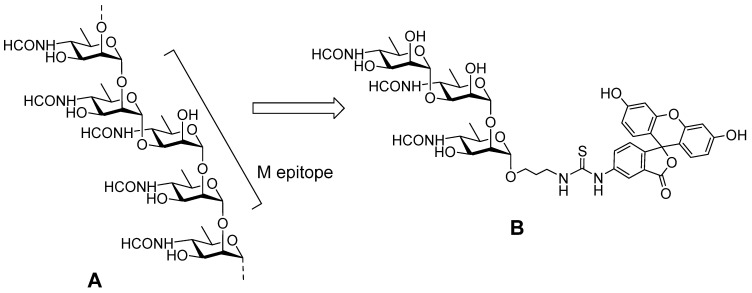
(**A**). Structure of the *Brucella* O-polysaccharide showing the M epitope. (**B**). Synthetic FITC-labelled biotracer used in diagnostic FPA tests for brucellosis detection.

Recent progress in the synthesis of antigenic spacer-armed oligosaccharides related to determinant fragments of carbohydrate chains exposed on the cell surface of pathogenic bacteria and fungi (for example, see [85,86,87,88,89,90,91]) represents a promising direction for the development of new FPA-based diagnostic protocols. This is already shown in the cases of screening of carbohydrate-specific antibodies [92,93] and the activity of the carbohydrate-dissecting enzyme lysozyme [94]. One of most recent examples of such developments is the FPA-based method for brucellosis diagnosis in animals [95]. It applies FITC-labelled tracer (Figure 6B) derived from a synthetic trisaccharide precursor of a distinct structure [96] which mimics the M-epitope part of the O-chain of *Brucella* O-polysaccharide (Figure 6A). Such a synthetic tracer for FPA has principal advantages when compared to above-described tracers on the basis of FITC-labelled bacterial O-polysaccharide, which is difficult to use in the preparation and standardization that is critical for reliable and reproducible analyses.

The advent of new portable devices for FPA enables the detection of infections directly at the POC, which also favors the expansion of the application of the discussed analytical method. It should also be mentioned that FPA can be useful not only in diagnosing diseases, but also in screening new drugs against viral, bacterial, and fungal pathogens, in monitoring the recognition of biological receptors, and in many other fields.

## Figures and Tables

**Table 1:** Sensitivity (Se) and specificity (Sp) of FPA for detection of antibodies to different infection agents.

Infectious Agent	Se	Sp	Cut-Off (*mP*)	Ref.
*B. abortus* and *B. melitensis*	88.7 99.0	92.5 95	89.9 90	[20] [34]
*B. melitensis*	95	100	74.1	[35]
*B. melitensis*	97.6	98.9	87	[36]
*B. suis*	93.5	97.2	84	[37]
*B. abortus* and *B. suis*	99	84	80	[38]
*M. bovis*	92.9	98.3	173.9	[39]
	96.9	64.6	127	[40]
*Paratuberculosis*	88.5	91.4	126	[24]
*Equine infectious anemia virus*	98	98	98.6	[21]

## Data Availability

The data are available from the corresponding author.
